# A new species of freshwater crab of the genus *Mediapotamon* Türkay & Dai, 1997 (Crustacea, Decapoda, Brachyura, Potamidae) from Guizhou, China

**DOI:** 10.3897/zookeys.873.36702

**Published:** 2019-08-29

**Authors:** Song-bo Wang, Xian-min Zhou, Jie-xin Zou

**Affiliations:** 1 Research Laboratory of Freshwater Crustacean Decapoda & Paragonimus, School of Basic Medical Sciences, Nanchang University, 461 Bayi Avenue, Nanchang City, Jiangxi Province 330006, China Nanchang University Nanchang China; 2 Key Laboratory of Poyang Lake Environment and Resource Utilization, Ministry of Education, Nanchang University, 1299 Xuefu Avenue, Nanchang City, Jiangxi Province 330031, China Nanchang University Nanchang China

**Keywords:** freshwater crab, *Mediapotamon liboense*, systematics, taxonomy, 16S rDNA

## Abstract

A new species of *Mediapotamon* Türkay & Dai, 1997 from a karst system in southwest China is described. The new species can be separated from congeners by the combination of a sharp and distinct epibranchial tooth, the anterolateral region lined with few scattered granules, the terminal segment of the male first gonopod distinctly bent with a constant diameter, and the position of the female vulvae. Mitochondrial 16S rDNA genetic data was used to investigate the systematic position of the new species, which is supported as a new taxon.

## Introduction

China has the highest number of freshwater crab species in the world, with more than 300 species (Dai 1999, Cumberlidge et al. 2011). There are unique karst landforms in Guizhou, China where numerous caves are distributed (Han et al. 2010), and researchers have discovered a number of new freshwater crab species in these caves (Ng and Trontelj 1996, Ng 2017, Huang et al. 2017). To investigate the species diversity of freshwater crabs in this area, the authors conducted scientific investigations twice in 2010 and 2017 to collect specimens of *Chinapotamon* Dai & Naiyanetr, 1994, *Mediapotamon* Türkay & Dai, 1997, *Daipotamon* Ng & Trontelj, 1996, and *Longpotamon* Shih, Huang & Ng, 2016, some of which have already been published (Shih et al. 2016). After morphological comparison of the collected specimens, the specimen from Yaozhai village, Dongtang town, Libo County, Qiannan Buyei and Miao Autonomous Prefecture, was found to be a new species of *Mediapotamon*. This new species is described in this paper, and although also distributed in the karst landforms, is found not in caves but in a hill stream between densely populated mountains. Individuals of this species do not have the characteristics of cave crabs, which determined it as not a karst species but also living in a surrounding karst system. We sequenced the mitochondrial 16S rDNA gene of a specimen and combined the sequence with related reference sequences in GenBank to establish a phylogenetic tree based on Bayesian Inference (BI) and Maximum Likelihood (ML) methods. The molecular data analysis was consistent with the morphological identification results, confirming that it is a new species.

## Materials and methods

Specimens were collected from Banzhai Hill, Yaozhai village (25.2128°N, 108.0041°E), Dongtang town, Lino County, Qiannan Buyei and Miao Autonomous Prefecture, Guizhou Province; preserved in 95% ethanol; and deposited at the Department of Parasitology of the Medical College of Nanchang University, Jiangxi, China (**NCU MCP**). Comparative materials were deposited at the Sun Yat-sen Museum of Biology, Sun Yat-sen University, Guangzhou, China (**SYSBM**) and the Institute of Zoology, Chinese Academy of Sciences, Beijing, China (**IZCAS CB**). Carapace width and length were measured in millimetres. The abbreviations **G1** and **G2** refer to the first and second gonopods, respectively. The terminology used herein primarily follows that of Dai (1999) and Davie et al. (2015).

Pereiopod muscle tissue was extracted from specimens of the new species with a DP1902 Tissue Kit (BioTeke Inc., Beijing). The mitochondrial 16S rDNA gene was obtained by PCR amplification with the primers 1471 (5’-CCTGTTTANCAAAAACAT-3’) and 1472 (5’-AGATAGAAACCAACCTGG-3’) (Shih et al. 2004). The PCR extension procedure is as follows: denaturation for 50 s at 94 °C, 33 cycles of annealing for 40 s at 52 °C and extension for 1 min at 72 °C and a final extension for 10 min at 72 °C. The PCR products were sequenced on an ABI 3730 automatic sequencer.

For molecular data analysis, the mitochondrial 16S rDNA from 52 species in 41 genera was used to construct a phylogenetic tree (Table 1). Sequences were aligned using MAFFT ver. 7.215 (Katoh and Standley 2013) based on the G-INS-I method. The best model for BI analysis was GTR+ I + G, which was determined by ModelGenerator ver. 8.5.1 (Katoh and Standley 2013) and the Bayesian information criterion (BIC). The BI tree was constructed by MrBayes ver. 3.2.6 (Ronquist et al. 2012). Four Markov chain Monte Carlo (MCMC) chains were run for 2000000 generations, with samples stored every 1000 generations, and the first 25% were discarded as burn-in. The effective sample size (ESS) values were checked by TRACER ver. 1.6 (Rambaut and Drummond 2013) (all ESSs were greater than 200). The best evolutionary model for ML analysis was HKY+I+G, as determined by MEGA 7.0 (Kumar et al. 2016) and ModelTest ver. 3.7 (David 2003) based on the Akaike information criterion (AIC) standard. A ML tree was built based on 1000 bootstrap replicates in MEGA 7.0 (Kumar et al. 2016).

**Table 1.** The 16S rDNA of 52 species from 41 genera of the family Potamidae from Asia. All sequences retrieved from GenBank except for the new species described herein.

**Species**	**Museum catalogue number**	**Locality**	**GenBank number**
*Amamiku amamense* (Minei, 1973)	NCHUZOOL 13125	Amami, the Ryukyus	AB428457
*Aparapotamon grahami* (Rathbun, 1929)	ZRC YCM 0334(II)	Yunnan, China	AB428489
*Apotamonautes hainanensis* (Parisi, 1916)	ZRC	Hainan, China	AB428459
*Beccumon jarujini* (Ng & Naiyanetr, 1993)	ZRC 1991.1865 (paratype)	Chiangma, Thailand	AB428479
*Candidiopotamon rathbunae* (De Man, 1914)	NCHUZOOL	Nantou, Taiwan	AB208598
*Chinapotamon glabrum* (Dai, Song, Li & Liang, 1980)	CAS	Guangxi, China	AB428451
*Chinapotamon maolanense* Zou, Bai & Zhou, 2018	NCU MCP 196101	Guizhou, China	11280060
*Cryptopotamon anacoluthon* (Kemp, 1918)	NCHUZOOL 13122	Hong Kong	AB428453
*Daipotamon minos* Ng & Trontelj, 1996	ZRC	Guizhou, China	LC198524
*Demanietta renongensis* (Rathbun, 1905)	ZRC 1998.146	Ranong, Thailand	AB428475
*Diyutamon cereum* Huang, Shih & Ng, 2017	SYSBM	Guizhou, China	LC198520
*Eosamon boonyaratae* (Naiyanetr, 1987)	ZRC 1991.1861	Trat, Thailand	AB428487
*Eosamon smithianum* (Kemp, 1923)	ZRC	Chantaburi, Thailand	AB428486 B428486
*Eosamon yotdomense* (Naiyanetr, 1984)	ZRC 1991.1851	Ubon Ratchathani, Thailand	AB428485
*Esanpotamon namsom* Naiyanetr & Ng, 1997	ZRC 1997.776 (paratype)	Udon Thani, Thailand	AB428463
*Flabellamon* sp.	ZRC	Mae Sot, Thailand	AB428472
*Geothelphusa albogilva* Shy, Ng & Yu, 1994	NCHUZOOL	Pingtung, Taiwan	AB127366
*Geothelphusa marginata fulva* Naruse, Shokita & Shy, 2004	NCHUZOOL 13124	Iriomote, the Ryukyus	AB428456
*Geothelphusa olea* Shy, Ng & Yu, 1994	NCHUZOOL 13123	Taichung, Taiwan	AB428455
*Hainanpotamon fuchengense* Dai, 1995	NCHUZOOL 13128	Hainan, China	AB428461
*Huananpotamon angulatum* (Dai & Lin, 1979)	ZRC	Fujian, China	AB428454
*Indochinamon ou* (Yeo & Ng, 1998)	ZRC	Phongsali, Laos	AB428481
*Indochinamon tannanti* (Rathbun, 1904)	ZRC 1998.264	Yunnan, China	AB428482
*Johora johorensis* (Roux, 1936)	ZRC 1990.576	Gunung Pulai, Johor, Malaysia	AB290620
*Johora murphyi* Ng, 1986	ZRC 2001.2267	Kota Tinggi, Johor, Malaysia	AB290621
*Kanpotamon duangkhaei* Ng & Naiyanetr, 1993	ZRC	Kanchanaburi, Thailand	AB428471
*Kukrimon cucphuongense* (Dang, 1975)	ZRC NHH9729 160997	Ninh Binh, Vietnam	AB428483
*Longpotamon baiyanense* Ng & Dai, 1997	ZRC	Hunan, China	AB428470
*Longpotamon planum* Dai, 1992	ZRC 1998.1178	Anhui, China	AB428469
*Mediapotamon leishanense* Dai, 1995	SYSBM001094	Guizhou, China	LC155164
*Mediapotamon liboense* sp. nov.	NCU MCP 343004	Guizhou, China	MK820377
*Mediapotamon liboense* sp. nov.	NCU MCP 343008	Guizhou, China	MK820376
*Mediapotamon* sp. nov., leg. Chao Huang	SYSBM001259	Guizhou, China	LC155165
*Megacephalomon kittikooni* (Yeo & Naiyanetr, 1999)	ZRC 1998.22 (holotype)	Xieng Khuang, Laos	AB428462
*Mindoron balssi* (Bott, 1968)	ZRC	Mindoro, the Philippines	AB428464
*Minpotamon nasicum* (Dai & Chen, 1979)	NCHUZOOL 13121	Fujian, China	AB428450
*Nanhaipotamon formosensis* (Parisi, 1916)	NCHUZOOL 13144	Tainan, Taiwan	AB212867
*Nanhaipotamon nanriense* Dai, 1997	CAS CB05103	Fujian, China	AB212868
*Neotiwaripotamon jianfengense* Dai & Naiyanetr, 1994	NCHUZOOL 13127	Hainan, China	AB428460
*Ovitamon artifrons* (Bürger, 1894)	ZRC	Luzon, the Philippines	AB428466
*Parapotamon spinescens* (Calman, 1905)	NCUDP	Yunnan, China	AB428467
*Pararanguna semilunatum* Dai & Chen, 1985	ZRC	Yunnan, China	AB428490
*Potamiscus yiwuensis* Dai & Cai, 1998	ZRC	Yunnan, China	AB428476
*Potamiscus yongshengense* Dai & Chen, 1985	NNU150951	Yunnan, China	KY963597
*Pudaengon sakonnakorn* Ng & Naiyanetr, 1995	ZRC	Thailand	AB428484
*Pupamon nayung* (Naiyanetr, 1993)	ZRC 1995.558 (paratype)	Udon Thani, Thailand	AB428477
*Ryukyum yaeyamense* (Minei, 1973)	NCHUZOOL 13126	Iriomote, the Ryukyus	AB428458
*Shanphusa curtobates* (Kemp, 1918)	NRM 13920	Taunggyi, Shan State, Myanmar	AB428478
*Socotrapotamon nojidensis* Apel & Brandis, 2000	ZRC 2000.2232	Socotra, Yemen	AB428493
*Tenuipotamon huaningense* Dai & Bo, 1994	CAS CB05175	Yunnan, China	AB428491
*Thaiphusa* sp.	ZRC 1997.656	Thailand	AB428474
*Tomaculamon pygmaeus* Yeo & Ng, 1997	ZRC 1997.326‐330 (paratype)	Phitsanulok, Thailand	AB428473
*Trichopotamon daliense* Dai & Chen, 1985	NCHUZOOL 13130	Yunnan, China	AB428492
*Yarepotamon gracilipa* (Dai, Song, Li & Liang, 1980)	ZRC	Guangxi, China	AB428452

Key to institutional abbreviations: CAS, The Chinese Academy of Sciences, Beijing, China; NCHUZOOL, Zoological Collections of the Department of Life Science, National Chung Hsing University, Taichung, Taiwan; NCUDP, Department of Parasitology, Nanchang University, Jiangxi, China; NRM, Swedish Museum of Natural History, Stockholm, Sweden; ZRC, Zoological Reference Collection of the Raffles Museum of Biodiversity Research, National University of Singapore, Singapore.

## Taxonomy

### Family Potamidae Ortmann, 1896

#### *Mediapotamon* Türkay & Dai, 1997

##### Mediapotamon liboense sp. nov.

Taxon classificationAnimaliaDecapodaPotamidae

CB41A406BBB755DBB21E7493007E7401

http://zoobank.org/69B0792B-F233-403A-ADC2-6666B007F093

**Figure 1.**
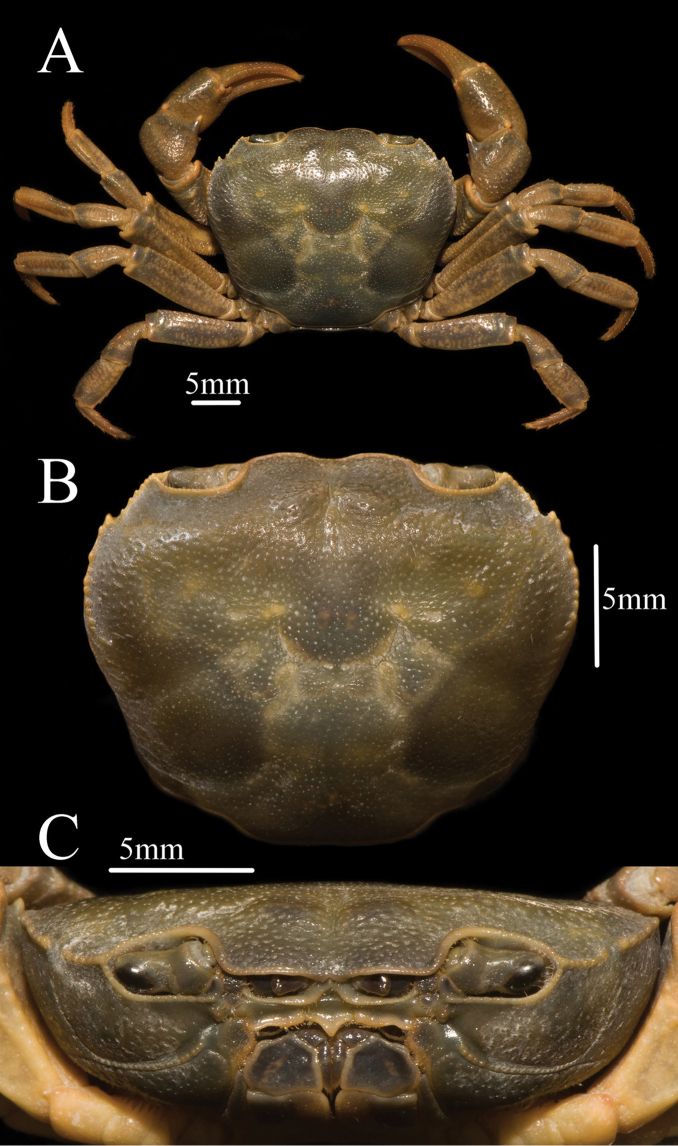
*Mediapotamon liboense* sp. nov. Holotype male (24.2 × 19.6 mm) (NCU MCP 343001). **A** overall habitus **B** dorsal view of carapace **C** frontal view of cephalothorax.

**Figure 2.**
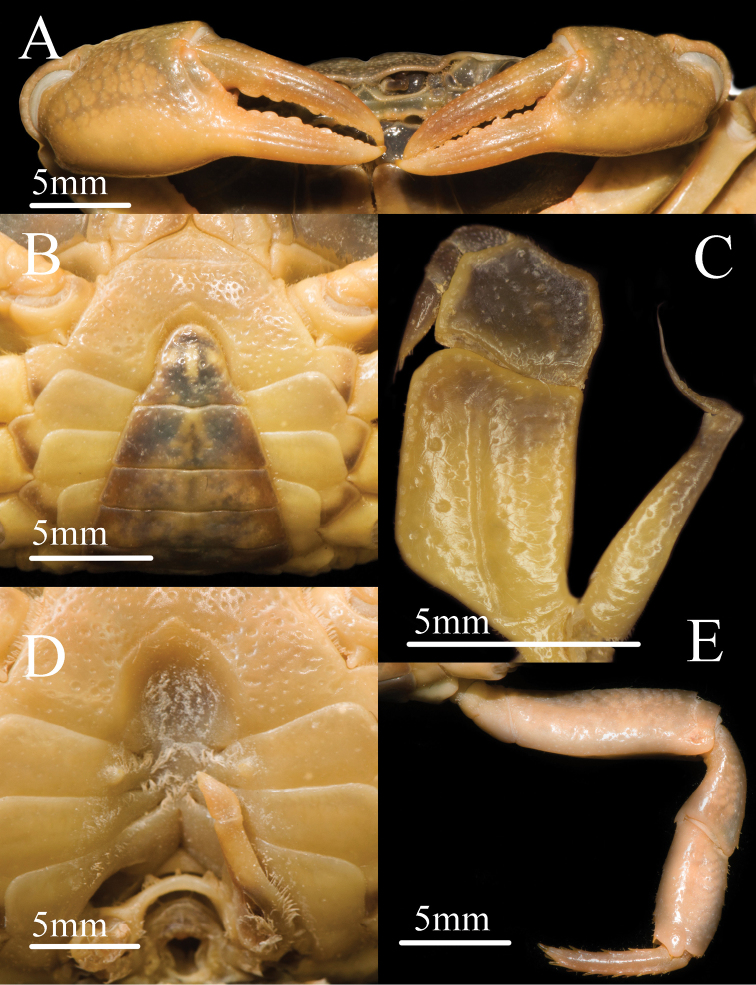
*Mediapotamon liboense* sp. nov. Holotype male (24.2 × 19.6 mm) (NCU MCP 343001). **A** outer view of chelipeds **B** ventral view of anterior thoracic sternum and pleon **C** left third maxilliped **D** ventral view of sterno-pleonal cavity with left G1 in situ **E** right fourth ambulatory leg, low view.

**Figure 3.**
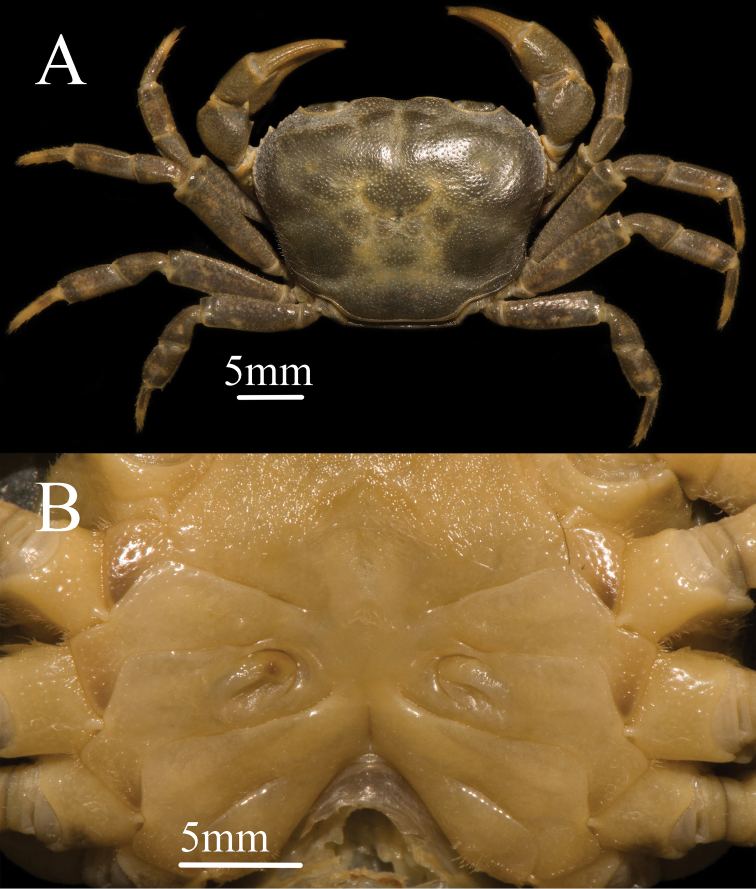
*Mediapotamon liboense* sp. nov. Paratype female (23.4 × 19.0 mm) (NCU MCP 343003). **A** overall habitus **B** ventral view of thoracic sternum and vulvae.

**Figure 4.**
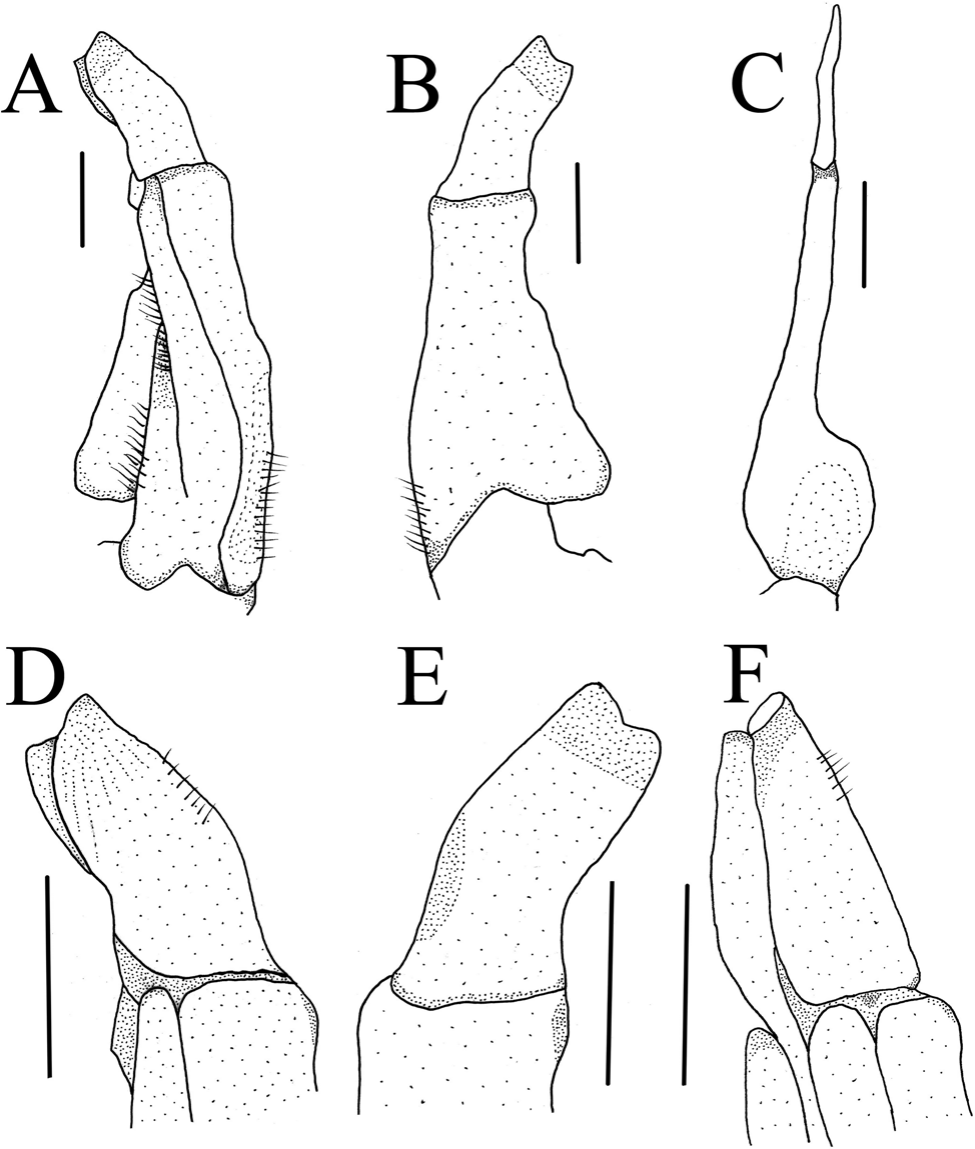
*Mediapotamon liboense* sp. nov. Holotype male (24.2 × 19.6 mm) (NCU MCP 343001). **A** ventral view of the left G1**B** dorsal view of the left G1**C** ventral view of the left G2**D** ventral view of the terminal segment of left G1**E** dorsal view of the terminal segment of left G1**F** mesial view of the terminal segment of left G1. Scale bars: 1 mm (**A–F**).

**Figure 5.**
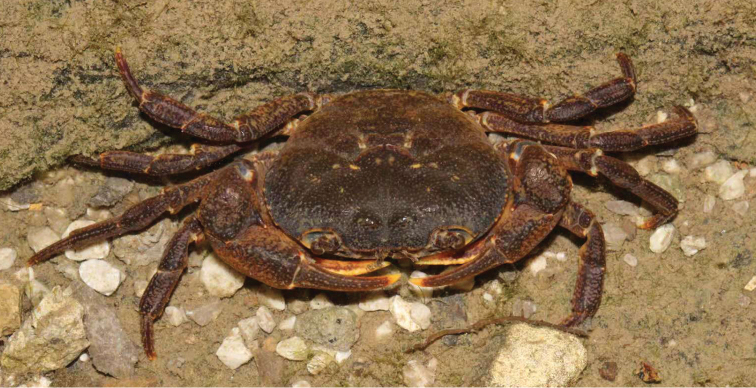
*Mediapotamon liboense* sp. nov. Colour in life, not collected (photograph by Chao Huang).

Figs 1 , 2 , 3 , 4 , 5

###### Type locality.

China, Guizhou Province: Qiannan Bouyei and Miao Autonomous Prefecture, Lino County, Dongtang Town, Yaozhai Village, Banzhai Hill, 25.2128°N, 108.0041°E, under rock in small hill stream.

###### Type specimen.

Holotype male, with gonopods in a separate microvial. Original label: “China, Guizhou Province: Qiannan Bouyei and Miao Autonomous Prefecture, Lino County, Dongtang Town, Yaozhai Village, Banzhai Hill, 25.2128°N, 108.0041°E, 10 Oct. 2010, Xian-min Zhou”, “NCU MCP 343001”. Paratypes, male, same collection data as for holotype, “NCU MCP 343002”; female, same collection data as for holotype, “NCU MCP 343003”.

###### Material examined.

**Holotype**. CHINA • ♂, NCU MCP 343001, 24.2 × 19.6 mm, Guizhou Province, Qiannan Bouyei and Miao Autonomous Prefecture, Lino County, Dongtang Town, Yaozhai Village, Banzhai Hill, under rock in small hill stream, catch by hand, 10 Oct 2010, Xian-min Zhou leg.

**Paratypes.** ♂, NCU MCP 343002,19.4 × 15.6 mm • ♀, NCU MCP 343003, 23.4 × 19.0 mm, same collection data as for holotype.

###### Other material.

♂, NCU MCP 343004, 30.9 × 24.7 mm • 4 ♀♀; NCU MCP 343005, 21.5 × 16.8mm; NCU MCP 343006, 19.2 × 14.6 mm; NCU MCP 343007, 25.0 × 20.0 mm; NCU MCP 343008, 20.0 × 15.8 mm; same collection data as for holotype.

###### Comparative material.

*Mediapotamon angustipedum* (Dai & Song, 1982): 2 ♂♂; IZCAS CB 00995, 15.3 × 13.1 mm; IZCAS CB 00988, 18.4 × 16.0 mm; Guangxi Zhuang Autonomous Region, Baise City, Jingxi County, Hurun Town, Xinxing Village, 7 Oct 1978. *Mediapotamon leishanense* (Dai, 1995): 1♂, IZCAS CB 05181, 14.8 × 11.5 mm, Guizhou Province, Qiandongnan Miao and Dong Autonomous Prefecture, Leishan County, Leigong Mountain, 23 Apr. 1988; 1♂, SYSBM 001094, 15.5 × 12.4 mm, Guizhou Province, Qiannan Bouyei and Miao Autonomous Prefecture, Lino County, coll. C. Huang, Jul 2013. *Mediapotamon* sp. nov. (sequence number LC155165 in Fig. 8): 1♂, SYSBM 001255, 26.7 × 21.4 mm, 1 ♀, SYSBM 001259, 17.5 × 13.6 mm, Guizhou Province, Qiannan Bouyei and Miao Autonomous Prefecture, Lino County, coll. C. Huang, Jul 2013. *Daipotamon minos* (Ng & Trontelj, 1996): 1♂, NCU MCP 195501, 20.1 × 16.3 mm, 1 ♀, NCU MCP 195502, 18.8 × 15.1 mm, Guizhou Province, Qiannan Bouyei and Miao Autonomous Prefecture, Lino County, Chaoyang Town, Buyong Village, coll. L. J. Yang, 17 Jul 2010.

###### Diagnosis.

Carapace trapezoidal, regions indistinct, dorsal surface flat, epigastric cristae indistinct, postorbital cristae convex, cervical groove indistinct, H-shaped groove inconspicuous (Figs 1A, B, 3A). External orbital angle triangular, epibranchial tooth sharp, anterolateral margin lined with scattered granules, posterolateral surface smooth (Figs 1A, B, 3A). Third maxilliped exopod with slender flagellum, extending equal to width of merus (Fig. 1C). Male pleon narrow triangular, telson with arc-shaped apex in male (Fig. 2B). G1 slender, terminal segment bend inwards obviously in sub-proximal portion with constant diameter, G1 terminal segment oblique toward dorsal in mesial view in the demarcation between G1/G2 (Figs 4A, F, 7A). Proximal part of G2 sub-ovate (Fig. 4C). Female vulva large-sized, not reaching suture sternites 5/6, vulval membrane extending outward (Fig. 3B).

###### Description.

**Carapace**: outline trapezoidal, width 1.2–1.3 × length (n = 8); dorsal surface flat with numerous pits, anterolateral region wrinkled (Figs 1A, B, 3A). Epigastric cristae indistinct; cervical groove shallow, indistinct; H shaped groove between gastric and cardiac regions inconspicuous (Figs 1A, B, 3A). Postorbital cristae slightly convex, not fused with epigastric cristae, separate with epibranchial tooth (Figs 1A, B, 3A). External orbital angle bluntly triangular, separate with anterolateral margin by conspicuous gap (Figs 1A, C, 3A). Epibranchial tooth sharp, distinct; anterolateral margin convex laterally, cristae, lined with approximately 9 or10 scattered granules (Figs 1A, B, 3A). Posterolateral surface smooth, with inconspicuous oblique striae, posterolateral margins converging posteriorly (Figs 1A, B, 3A). Orbits medium-size; supraorbital margin cristate and lateral portion, infraorbital margins lined with scattered inconspicuous granules (Fig. 1C). Sub-orbital, sub-hepatic and pterygostomial regions covered with low round granules (Fig. 1C). Epistome posterior margin slightly oblique laterally, with broadly triangular median lobe (Fig. 1C).

**Third maxilliped**: exopod reaching proximal 1/3 of merus length, with slender flagellum extending equal to width of merus (Figs 1C, 2C). Merus subquadrate, 1.3 times as broad as long, generally flat (Figs 1C, 2C). Ischium trapezoidal, 1.4 times as long as broad, with distinct median sulcus (Fig. 2C).

**Chelipeds (pereiopod 1)**: slightly unequal (Fig. 2A). Merus surface smooth; carpus surface with pits and a sharp spine at inner-distal angle (Figs 1A, 3A). Palm of larger chela length 1.4 × height in males (n = 3), 1.5–1.6 × in females (n = 5); dactylus 1 × palm length in males (n = 3), 0.9–1 × in females (n = 5); dactylus as long as pollex (Figs 1A, 2A, 3A). Inner margin of fingers with few round blunt teeth, with little gap when fingers closed (Fig. 2A).

**Ambulatory legs (pereiopods 2–5)**: slender; pereiopod 3 merus 0.5 × carapace length in males (n = 3), 0.4 × carapace length in females (n = 5) (Figs 1A, 3A). Pereiopods 5 propodus 1.9 × as long as broad in males (n = 3), 1.9–2.1 × as long as broad in females (n = 5) (Fig. 2E); shorter than dactylus (n = 8) (Figs 1A, 2E, 3A).

**Male thoracic sternum**: flat and covered with pits; sternites 2-4 broad, width ca. 2 × length; sternites 2 very broad triangular with sharp apex; suture between sternites 2/3 transverse, clear; sternites 3/4 fused but with slight oblique demarcation superficially (Fig. 2B). Male sterno-pleonal cavity deep and narrow, barely reaching anteriorly to level of mid-length of cheliped coxae base; median longitudinal groove present between sternites 7 and 8 medium in length; male pleonal locking tubercle position at middle of sternite 5 (Fig. 2D).

**Male pleon**: narrow triangular (Fig. 2B); somites 4-6 progressively narrowed distally, lateral margins oblique; telson width 1.3 × length with arc-shaped apex in males (n = 3); somite 6 width 2.4 × length in males (n = 3) (Fig. 2B).

**G1**: slender (Figs 4A, 7A); terminal segment bend inwards obviously in the sub-proximal portion with constant diameter, distal end reaching but not beyond pleonal locking tubercle *in situ* (Fig. 2D); subterminal segment length 2.9 × length of terminal segment (Figs 4A, 7A). The mesial view of G1 terminal segment not straight but oblique toward dorsal in the demarcation between G1/G2 (Fig. 4F). Basal segment of G2 sub-ovate, subterminal segment length 1.8 × length of distal segment, the distal segment is slender and sharp (Fig. 4C).

**Female vulva**: large, not reaching sternites 5/6 in situ, with the opening outward and the outer membrane extending outward (Fig. 3B). Reaching approximate three-fifths width of sternite 6 and the position generally distantly each other (Fig. 3B).

###### Etymology.

The species is named after the type locality, Libo County, Qiannan Bouyei and Miao Autonomous Prefecture, Guizhou Province.

###### Colour in life.

The overall colour is brownish black, which is similar to the colour of the surrounding environment (Fig. 5).

###### Distribution.

The new species is presently known only from the type locality: Libo County, Qiannan Bouyei and Miao Autonomous Prefecture, Guizhou Province.

###### Ecology.

**Figure 6.**
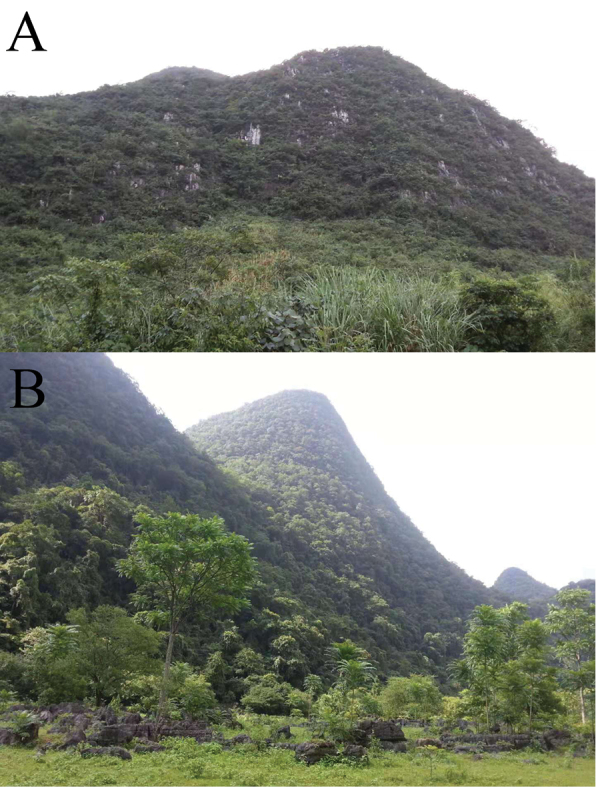
Karst terrain of Libo County (photographs by Chao Huang).

This species lives in karst mountain locations surrounded by low crests and covered with diverse vegetation (Figs 6A, B). The species lives along the stream flowing down the mountain and remains hidden under rocks during the day.

###### Remarks.

**Figure 7.**
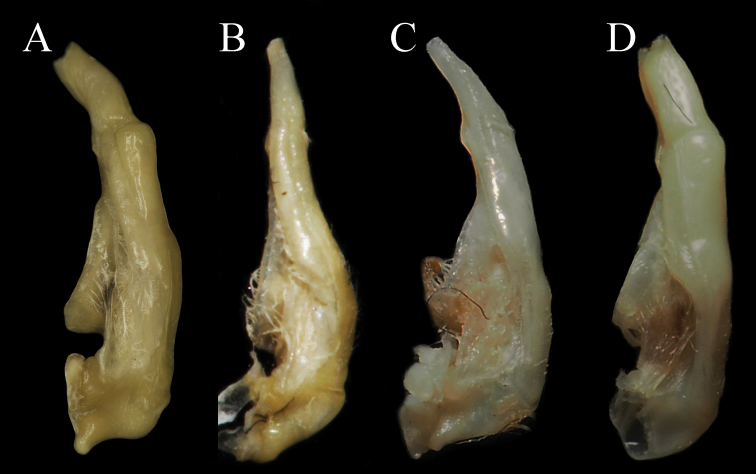
Left G1s. **A***Mediapotamon liboense* sp. nov. NCU MCP, 24.2 × 19.6 mm **B***M. angustipedum* (Dai & Song, 1982), IZCAS CB 00995, 15.3 × 13.1 mm **C***M. leishanense* Dai, 1995, IZCAS CB 05181,14.8 × 11.5 mm **D***Daipotamon minos*, Ng & Trontelj, 1996, NCU MCP 195501, 20.1 × 16.3 mm.

The new species fits the characteristics of *Mediapotamon* Türkay & Dai, 1997, viz., carapace intermediate or small in size (15–20 mm), surface smooth without a conspicuous bulge or depression, anterolateral margin lined with granules, male telson triangular, and G1 slender without any projection and reaching the pleonal locking tubercle *in situ* (Türkay and Dai 1997). *Mediapotamon liboense* sp. nov. is similar to *M. angustipedum* (Dai & Song, 1982), *M. leishanense* Dai, 1995, and *Daipotamon minos*, Ng & Trontelj, 1996, but the new species can be differentiated from its congeners by some distinct characters: epibranchial tooth sharp and distinct, anterolateral margin lined with a few scattered granules [versus sharp and distinct in *M. angustipedum* but blunt and indistinct in *M. leishanense*, both lined with numerous inseparable granules (cf. Table 2)]; shape of the male telson narrow triangular [versus broad triangular in congeners (cf. Table 2, Fig. 7)]; and shape of G1 slender, terminal segment distinctly bent with a constant diameter [versus very slender, terminal segment straight and thinner gradually in *M. angustipedum* and very slender, terminal segment bent obviously and thinner gradually in *M. leishanense* (cf. Table 2, Fig. 7)]. Its differences compared to *D. minos* can be found in Table 2 and Figure 7.

## DNA analyses and discussion

**Figure 8.**
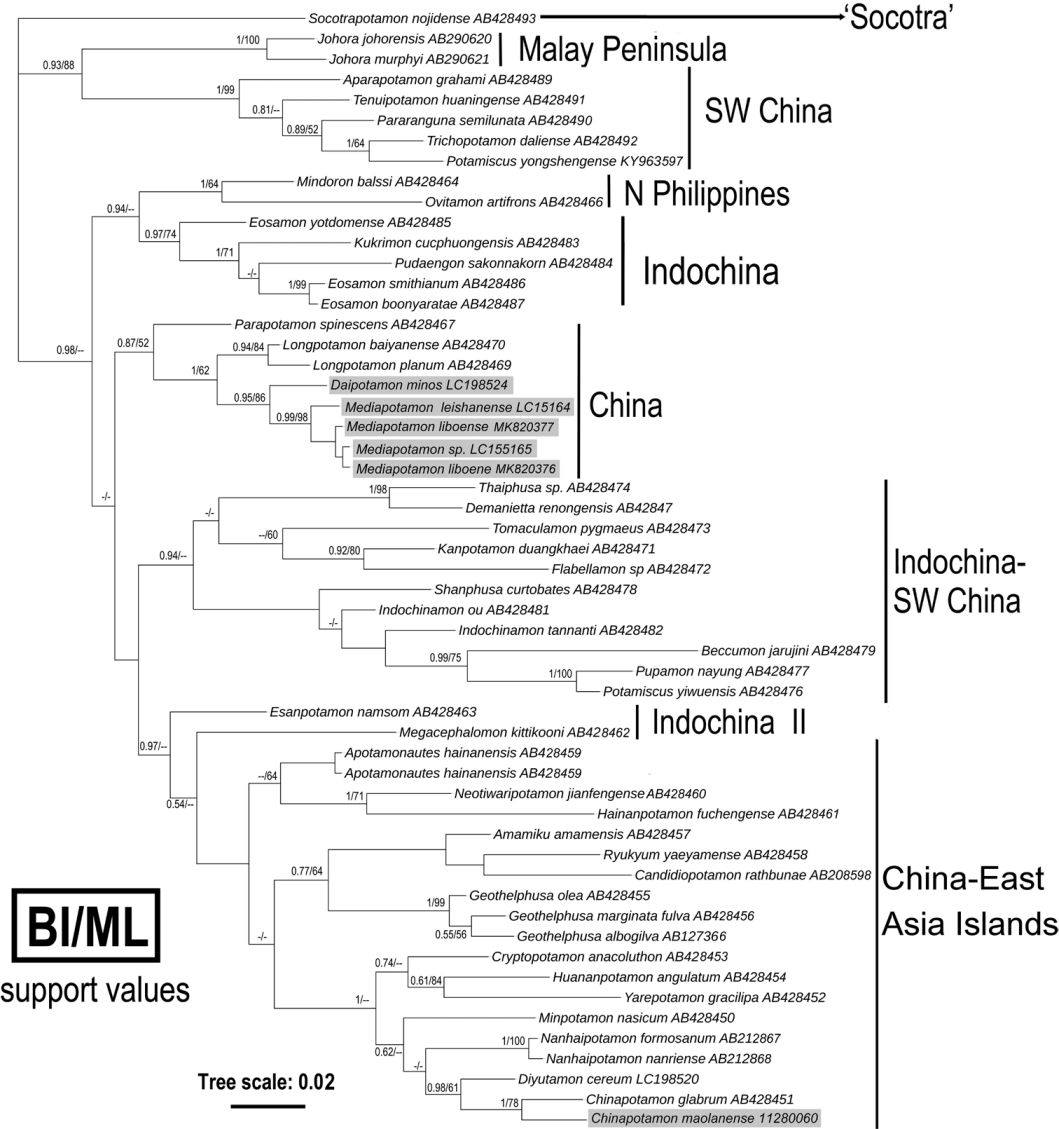
A Bayesian inference (BI) tree based on 16S rDNA with the sequences and accession numbers from Shih et al. (2009) with some additional species from Guizhou. The species collected from the type locality and its surroundings are highlighted in grey. The probability values at the nodes represent support values for BI and maximum likelihood (ML). Only values > 50% are displayed.

We used the mitochondrial 16S rDNA gene sequence for phylogenetic analyses, and 52 species from 41 potamid genera were included (Table 1), using BI and ML analyses to construct phylogenetic trees with support values. The results are shown in Figure 8, and both analysis methods support most of the clades (Shih et al. 2009). The new species clusters with the same species as *M. liboense* and *M. leishanense* (specimen collected by Chao Huang in the Maolan Nature Reserve of Libo County in July 2013). After discussion with Huang, we think that the other new species of *Mediapotamon* with sequence number is LC155165 and *M. liboense* sp. nov. are the same species, although the two specimens were collected separately. In the phylogenetic tree, *Daipotamon* is clustered with *Mediapotamon* in two separate branches, and the phylogenetic relationships between the new species and *Chinapotamon maolanense*, which was also collected in Libo County, are distant (Fig. 8)

Despite the new species clustering with congeners and *Daipotamon* in the larger clade, the genetic distance suggests that the congeners are closer, while *Daipotamon* is farther away. For the habitat, *Daipotamon* lives in limestone formations and collected from one of karst caves and was determined as a karst species (Ng and Trontelj 1996), while the new species lives in hill streams, which is consistent with congeners, so the new species can be separated from *Daipotamon* in morphology, phylogenetic analyses, and ecology (Table 2, Fig. 8). *Mediapotamon* contains *M. leishanense* and *M. angustipedum* (Dai 1999), but we were unable to obtain molecular data for the latter, so its phylogenetic relationship with the new species is unclear. From molecular and morphological data, it is distinct from *M. leishanense*. Although there is no molecular data for *M. angustipedum*, the distinct morphological differences and more than 400 kilometres geographical distance separate the new species from *M. angustipedum* clearly. Morphological differences among the three *Mediapotamon* species, including the new species described in this study, are described in detail (Table 2).

**Table 2.** Differences between *Mediapotamon liboense* sp. nov., *M. angustipedum* (Dai & Song, 1982), *M. leishanense* Dai, 1995 and *Daipotamon minos*, Ng & Trontelj, 1996.

Character/ Species	*M. liboense* sp. nov.	*M. angustipedum*	*M. leishanense*	*Daipotamon minos*
Carapace	Flat, cervical groove indistinct	Swollen, cervical groove indistinct	Flat, cervical groove distinct	Slightly swollen, cervical groove distinct
Epibranchial tooth	Sharp, distinct	Sharp, distinct	Blunt, indistinct	Blunt, indistinct
Anterolateral margin	Lined with scattered granules	Lined with numerous inseparable granules	Lined with numerous inseparable granules	Lined with numerous inseparable granules
Shape of male telson	Narrow triangular	Broad triangular	Broad triangular	Tongue-shape
G1 *in situ*	Reaching pleonal locking tubercle	Reaching pleonal locking tubercle	Reaching pleonal locking tubercle	Not reaching pleonal locking tubercle
Shape of G1	Slender, terminal segment obviously bent with constant diameter	Very slender, terminal segment straight and gradually narrowing	Very slender, terminal segment obviously bent and gradually narrowing	Stout, terminal segment slightly bent with constant diameter
Female vulvae	Large-sized, not reaching sternites 5/6 in situ	Medium-sized, reaching sternites 5/6 in situ	Large-sized, reaching sternites 5/6 in situ	Medium-sized, not reaching sternites 5/6 in situ

Before our study, only three new species, namely *Diyutamon cereum*, *Qianguimon elongatum*, and *Chinapotamon maolanense*, collected in Guizhou had been published in the past 20 years (Huang et al. 2017, Huang 2018, Zou et al. 2018), implying that the freshwater crabs in this area still have high taxonomic research value. With the new species presently described, there are now 31 species of 13 potamid genera in Guizhou (Shih and Ng 2011).

## Supplementary Material

XML Treatment for Mediapotamon liboense
